# Comparative content analysis of national health policies, strategies and plans before and after COVID-19 among OECD and BRICS countries

**DOI:** 10.1186/s41256-024-00400-y

**Published:** 2025-02-21

**Authors:** Jialu Song, Ziqi Zhu, Qi Li, Ying Chen, Zhebin Wang, Shuduo Zhou, Ming Xu, Zhi-Jie Zheng

**Affiliations:** 1 Peking University First Hospital, No.8 Xishiku Road, Xicheng District, Beijing, 10034 China; 2 Peking University School of Public Health, 38 Xue Yuan Road, Haidian District, Beijing, 100191 China; 3 Institute of Social Development, Chinese Academy of Macroeconomic Research, Beijing, China; 4 Institute for Global Health and Development, Peking University, Beijing, China

**Keywords:** Policy content analysis, National health policy, Strategy or plan, System strengthening, COVID-19

## Abstract

**Background:**

The COVID-19 pandemic exposed significant limitations in health systems worldwide and emphasized the need for updated National Health Policies, Strategies, and Plans (NHPSPs). This study aimed to evaluate the NHPSPs of Organization for Economic Cooperation and Development (OECD) and BRICS (Brazil, Russia, India, China, and South Africa) countries before and after the COVID-19 pandemic. Specifically, it explored each country’s commitment to strengthening health systems to address health threats and analyzed the specific changes made.

**Methods:**

NHPSP documents from the WHO document repository and official governmental websites were systematically searched. Data were extracted using a standardized template. A coding framework was inductively developed to sort qualitative texts into categories, with frequencies calculated and weighting evaluated, followed by organizing underlying content into subthemes.

**Results:**

Out of 154 documents initially identified, 36 met the screening criteria, covering 14 OECD and 3 BRICS countries. The most predominant theme was prevention (88.9% pre-pandemic, 99.4% post-pandemic), which was addressed as a primary theme in 26 included NHPSPs. After the COVID-19 pandemic, 6 out of 14 analyzed themes saw higher occurrences, among which infection prevention and control (22.2–50.0%) and resilience to health crisis (22.2–44.4%) increased most significantly. Themes mainstreamed in post-pandemic NHPSPs included prevention (94.4%), health research and technology (61.1%), and One Health (66.7%). Primary healthcare emerged as the most concerned subtheme under prevention. Notably, OECD countries displayed more increased occurrences of themes (13 out of 14) or increased emphasis on themes with similar occurrences before and after COVID-19, while BRICS countries only differed in infection control. Additionally, OECD and BRICS countries varied in their subthemes and specific actions under similar primary themes.

**Conclusions:**

COVID-19 exposed vulnerabilities in many countries' health systems, highlighting the need to build resilient health infrastructures through the optimization of NHPSPs. However, only about half of the OECD and BRICS countries have implemented new NHPSPs since the pandemic. Our findings highlight the critical need for global health system reforms and offer actionable recommendations for other countries in formulating their NHPSPs.

**Supplementary Information:**

The online version contains supplementary material available at 10.1186/s41256-024-00400-y.

## Introduction

National health policy, strategy or plan (NHPSP) serves as a blueprint that guides the regulation and operation of the health system at both national and regional levels. Consequently, NHPSPs play a crucial role in shaping the health and well-being of populations, especially amid growing public expectations and demands. As a crucially important part of NHPSPs, health priorities must be determined to strategize the allocation of limited domestic resources optimally. NHPSPs, including health priorities, require sensitive tailoring to national needs due to diverse political, technical, and health systems characteristics within and across countries over time [1]. This diversity means there is rarely a "one-size-fits-all" solution. According to the World Health Organization, effective health priority-setting involves five critical criteria, including the burden of health issues, effectiveness of the intervention, cost of the intervention, acceptability of the intervention, and fairness [2]. Therefore, developing NHPSPs and health priorities is a complex and dynamic process, which varies from country to country due to political, social, historical and socio-economic factors, and it must be compatible with the major health issues.

Health security and health development are two important issues of NHPSPs. The World Health Organization (WHO) declared COVID-19 as a Public Health Emergency of international concern on January 30, 2020, and a pandemic on March 11, 2020 [3, 4]. By February 25, 2024, approximately 774 million COVID-19 cases and 7 million deaths had been reported to WHO [5]. The acute COVID-19 pandemic serves as a wake-up call to pause and reflect on whether the catastrophic impacts of this unprecedented health crisis were a contingency or an inevitable consequence of the weaknesses of health systems of countries. The pandemic revealed multiple vulnerabilities in health systems, including weak surveillance and reporting systems, poor coordination, insufficient financing and workforce, health inequalities, and limited healthcare capacity [6, 7].

As the world begins to move past the pandemic, building robust, resilient, and people-centered health systems is essential to prepare for future health challenges. Health system resilience is not just about mitigating damage and heal, but to thrive, by meeting the evolving needs of vulnerable populations, promoting social connectedness and address the underlying social determinants of both physical and psychological health [8, 9]. Achieving this resilience relies heavily on a structural health reform guided by top-level policy design. However, most existing literature only evolved around NHPSPs targeting specific diseases. In response to COVID-19, the WHO commission recommended that policymakers lay great emphasis on "One Health"(the health of humans, animals, and the environment) [10]. At the same time, other studies focused on specific areas regarding COVID-19 such as specific populations including children and women or particular domains such as health resilience, vaccine and healthcare workforce [9, 11–13]. Additional research has concentrated on areas like musculoskeletal health, non-communicable diseases, and cancer, but lacks a comprehensive analysis and comparison across countries [14–19]. Moreover, there is a significant knowledge gap regarding national-level policy comparisons before and after the COVID-19 pandemic, particularly in light of the changes to the International Health Regulations in May 2024 and ongoing discussions about a pandemic treaty.

To fill these gaps, this study aimed to identify and analyze the content of NHPSPs in selected countries before and after COVID-19, especially in preventing and managing health threats. Our findings are expcted to underscore the necessity for global health system reforms and provide other countries with actionable, sustainable policy recommendations for preventing and responding to future crises, thereby setting health systems and societies on a stable path for future generations.

## Methods

### Design

To cover a representative sample of countries, OECD and BRICS countries were chosen, representing high-income and low-and-middle-income countries, respectively. A systematic comparative content analysis of NHPSP documents of OECD and BRICS countries before and after the outbreak of the COVID-19 pandemic were undertaken (38 OECD countries including Australia, Austria, Belgium, Canada, Chile, Colombia, Costa Rica, Czech Republic, Denmark, Estonia, Finland, France, Germany, Greece, Hungary, Iceland, Ireland, Israel, Italy, Japan, Korea, Latvia, Lithuania, Luxembourg, Mexico, Netherlands, New Zealand, Norway, Poland, Portugal, Slovak Republic, Slovenia, Spain, Sweden, Switzerland, Türkiye, United Kingdom, and United States; 5 BRICS countries including Brazil, Russia, India, China, and South Africa). Our study focuses on the original five BRICS countries as these nations have been part of the BRICS framework for a significant period, allowing us to analyze their national health policies both before and after the COVID-19 pandemic in a consistent manner. The newly admitted BRICS countries in 2024 were not included in this study because their recent admission occurred after the period of data collection and analysis, and their inclusion would not provide sufficient historical data for a meaningful comparison within the scope of our research.

### Document search and selection

Using the following strategies, NHPSP documents were systematically searched. First, the researchers extracted documents from the WHO Country File Repository of the Country Planning Cycle Database. The database provides a country-by-country overview of national planning, health programmatic, and project cycles. Second, publications were searched from each country's official governmental websites or health ministry web pages. Third, a systematic desktop internet search was conducted on Google using the terms "national health policy, strategy or plan" in combination with the names of the countries to avoid omission.

The eligibility of NHPSP documents was assessed independently by two reviewers against the following inclusion criteria: national-level health policy, strategy, or action plan, published by the national government, health-related ministries, governmental institutions or parliaments, and in language that could be translated effectively into English using the online translation tool. The two reviewers then checked whether each country had eligible NHPSP documents before and after the COVID-19 pandemic; if not, the country would be excluded. Pre-pandemic NHPSPs were considered to be documents published before 2019 or published after 2019 but formulated before COVID-19, while post-pandemic NHPSPs were defined as documents that mentioned COVID-19 in any sections. If more than one document met the inclusion criteria, the most recent document of each country was included. Any disagreement was resolved by consensus meeting, and where two reviewers could not meet an agreement, the final decision was made after discussion among all the co-authors.

### Data extraction

A data extraction template for NHPSP was pre-established, drawing upon the WHO National Health Strategy Handbook, which offers comprehensive and practical guidance on national health planning and strategy development, including situation analysis, priority setting, and strategic planning (see online Appendix 1) [19]. Additionally, the researchers integrated a standardized data extraction template from a prior policy analysis that was based on the WHO Health Strategic Handbook and was tailored specifically to policy analysis [20]. The data extraction template collected data on publication information, policy background, purpose, aim or vision of the policy, health priorities or themes, major health issues or burdens, objectives, and specific strategies/action plans proposed to achieve these objectives and implementation mechanisms. Two reviewers independently extracted data from all selected NHPSP documents using the template, then integrated the extraction results with divergence solved by discussion among all authors. Documents published in non-English languages were translated using dual online translation software, Google Translate and Youdao Translate, to avoid mistranslation. Afterward, the data extraction sheet was quality-checked by the corresponding author, who holds a background in health policy research and has extensive experience in conducting systematic reviews and policy analysis.

### Data analysis and synthesis

Summative analysis was applied to extensive data, including policy background, content, and implementation mechanism. At the same time, purpose, aim or vision and priorities were reported as descriptive text excerpts using established methods for content analysis [21]. A three-step process was undertaken to analyze health priorities. First, one reviewer inductively developed a coding framework (first-order codes) based on the documents to sort qualitative texts into categories. Second, the coding framework was verified by the other two reviewers, with discrepancies resolved through discussion. Third, after the health priorities were sorted into 14 themes for comparative study, the frequencies of these themes were calculated as the number of documents in which they occurred, and weighting was estimated through seeking the part of the document where the themes appeared, such as a whole primary chapter, a minor chapter or just been mentioned, followed by the detailed interpretation of the underlying context. A similar approach was applied to the analysis of policy background and implementation mechanism.

## Results

### Overview of included NHPSPs

**Fig. 1:**
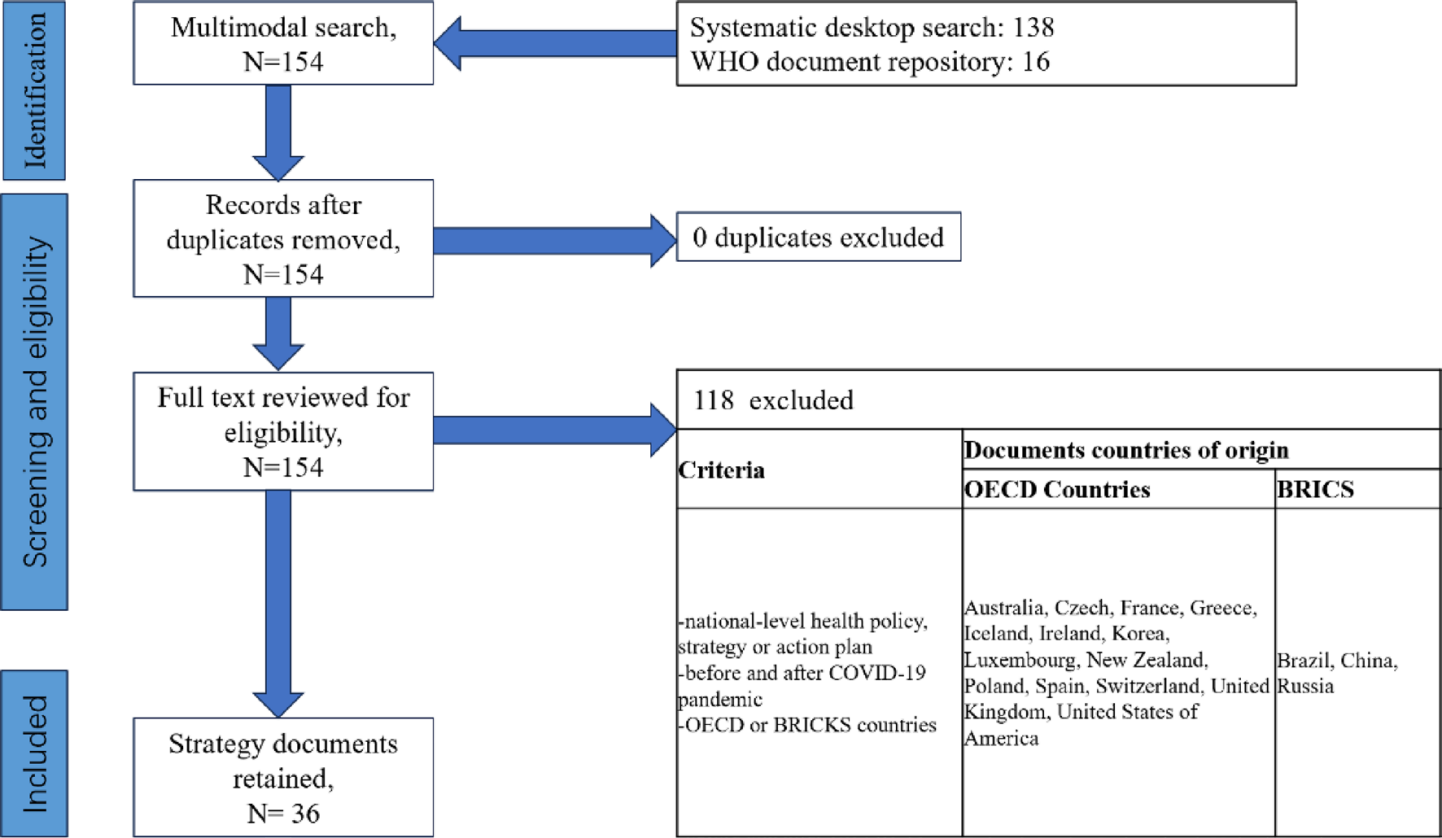
Comparative content analysis of NHPSPs before and after COVID-19

154 potentially eligible documents of OECD or BRICS countries were identified through a systematic desktop search (n = 138) and WHO document repository (n = 16). 0 was excluded as duplicates. 118 documents did not meet the inclusion criteria. After exclusions, 36 documents remained for analysis, including 14 OECD countries (Australia, Czech, France, Greece, Iceland, Ireland, Korea, Luxembourg, New Zealand, Poland, Spain, Switzerland, United Kingdom and United States of America), and 3 BRICS countries (Brazil, China and Russia) (see Fig. 1, Table 1). Each country has a pair of pre-and post-pandemic NHPSPs, except the United States, which has 2 pairs: Department of Health and Human Services (HHS) Strategic Plan and Healthy People. Most of the NHPSPs included focus on the overall health system. However, only two post-pandemic NHPSPs targeting at COVID-19 in Switzerland and Russia were found and used as substitutes for the overall-health-system NHPSPs that were not available. The purpose, aim, or vision of each NHPSP was described verbatim (see Table 1).

**Table 1:** List of policy documents by counties, title, year of publication, and vision

Country	Title	Year published (years operational)	Purpose, aim or vision
Australia	Australia: the healthiest country by 2020 national preventative health strategy – the roadmap for action	2009(2009–2020)	This Strategy sets out a vision for Australia to be the healthiest country by 2020
Australia	National preventive health strategy 2021–2030	2021(2021–2030)	To improve the health and wellbeing of all Australians at all stages of life through prevention
Brazil	Plano Nacional de Saúde PNS 2020–2023(national health plan 2020–2023)	2020(2020–2023)	Promote the health and well-being of all people through the development and implementation of public health policies, guided by universality, integrity and equity
Brazil	Plano Nacional de Saúde 2020–2023 national health plan 2020–2023	2021(2020–2030)	An effective public health system is recognized by all
China	“健康中国2030”规划纲要 the health China 2030 plan	2019(2020–2030)	Promote the building of a healthy China and improve the people's health By 2030, the system for promoting universal health will be improved, the health sector will be more coordinated, healthy lifestyles will be popularized, the quality of health services and health security will be improved, the health industry will flourish, health equity will be basically achieved, and major health indicators will become high-income countries
China	“十四五”国民健康规划; The 14th five-year national health plan	2022(2021–2025)	In 2035, the establishment and implement basic health care system that can meet the needs of socialist modernization, the basic medical and health system with Chinese characteristics to be more perfect, life expectancy of 80 years of age or older, per capita health gradually increase life expectancy
Czech Republic	Health 2020 – national strategy for health protection and promotion and disease prevention	2014(2014–2020)	The aim of the strategy is to stabilize the system of disease prevention, health protection and promotion and to initiate efficient mechanisms to improve health of the population, sustainable in the long-term The main strategic objective of the National Strategy is improving health of the population and reducing preventable diseases and premature deaths. Vision 2020 includes stabilizing disease prevention, health protection and promotion systems, establishing structures and mechanisms that enable collaboration between sectors and involving public sector at all levels in health improvement. Supporting good health leads to improving well-being and quality of life, a more efficient workforce, reducing healthcare expenditure, and increasing healthy life expectancy Improving health of the population and reducing preventable diseases and premature deaths
Czech Republic	Health 2030 – strategic framework for healthcare development in the Czech republic until 2030	2020(2021–2030)	By adopting the Czech Republic 2030 Strategic Framework, the government of the Czech Republic has identified a basic overarching goal for the health sector, which is “The health of all population groups is improving.”
France	Stratégie nationale de santé 2018–2022(National health strategy 2018–2022)	2018(2018–2022)	
France	Stratégie nationale de santé 2023–2033(national health strategy 2023–2033)	2023(2023–2033)	The HCSP defines four broad medium-and long-term objectives:1. Rebuild the health system to meet the challenges. 2. Reduce the impact of chronic diseases. 3. Strengthen actions towards the most vulnerable. 4. Implement a systemic prevention policy that addresses the main determinants of health using a population approach
Greece	Εθνική Στρατηγική Υγείας και δράσεις του τομέα υγείας στο ΕΣΠΑ 2014–2020	2014(2014–2020)	The vision of the Health Strategy in NSRF 2014–2020 is determined by the need for: Universal health coverage of the population, elimination of inequalities in the field of Health, while ensuring the conditions of sustainability of the National Health System
Greece	Ε ΘΝΙΚΟ Σ ΧΕΔΙΟ Δ ΡΑΣΗΣ Δ ΗΜΟΣΙΑΣ Υ ΓΕΙΑΣ 2021–2025 national action plan public health 2021–2025	2021(2021–2025)	The National Public Health Strategy is based on the basic policy vision of the overall strategy of the Ministry of Health: The elimination of health risk factors (socio-economic, behavioral and environmental). By extension, the fundamental policy objective of Public Health is formulated as: the continuous improvement of the level of health and the upgrading of the quality of life of the population, through the promotion of cross-sectoral cooperation to safeguard Public Health
Iceland	Health policy-A policy for Iceland’s health services until 2030	2019(2020–2030)	The following future vision is hereby laid down for the Icelandic health system, the guiding principle being that the people of Iceland should have reliable and efficient health services to which everyone is guaranteed access: Iceland’s health services stand comparison with the best in the world. Public health work focuses on the promotion of health and preventive measures play a part in all services, particularly those of the primary health clinics. The health services’ performance is assessed by measuring the quality of services, their safety, their accessibility and their cost
Iceland	Fimm ára aðgerðaáætlun heilbrigðisstefnu 2021 til 2025 five-year health policy action plan 2021–2025	2020(2021–2025)	The Vision and Strategy for 2030 consists of the following items: Icelandic healthcare is world-class and public health work focused on health promotion and prevention is part of all services, especially healthcare services
Ireland	Healthy Ireland-a framework for improved health and wellbeing 2013–2025	2013(2013–2025)	A Healthy Ireland, where everyone can enjoy physical and mental health and wellbeing to their full potential, where wellbeing is valued and supported at every level of society and is everyone’s responsibility
Ireland	The health service executive health protection strategy 2022–2027	2022(2022–2027)	The health and wellbeing of people in Ireland is protected against all health protection hazards
Luxembourg	OECD health policy overview-health policy in Luxembourg	2017	
Luxembourg	Plan national santé; national health plan	2023(2023–2033)	The ambition of the National health Plan is to develop the Luxembourg health system towards a system that is sustainable and equitable and at the forefront of progress; A system in which the citizen has confidence and in which health professionals are proud to be involved. In this way the health system will meet the criteria of the welfare economy, as defined by the who, and contribute to human, social, economic and global well-being
New Zealand	Health policy in New Zealand	2017	
New Zealand	New Zealand health strategy 2023	2023(2023–2033)	Our long-term vision is to achieve pae ora | healthy futures for all New Zealanders The New Zealand Health Strategy’s vision of pae ora is underpinned by two long-term goals. These are: To achieve health equity for our diverse communities, and especially for Māori, Pacific, disabled and other groups who currently have poorer outcomes To improve health outcomes for all New Zealanders
Poland	Narodowy Program Zdrowia Na Lata 2016–2020(national health plan 2016–2020)	2016(2016–2020)	The strategic objective of the National Health Programme for 2016–2020, hereinafter referred to as the "NPZ", is to extend healthy life, improve health and the related quality of life of the population, and reduce social inequalities in health
Poland	Narodowy Program Zdrowia na lata 2021–2025(national health programme for 2021–2025)	2021(2021–2025)	The strategic objective of the National Health Programme for 2021–2025, hereinafter referred to as the "NPZ", is to increase the number of years lived in health and to reduce social inequalities in health
Republic of Korea	제4차 국민건강증진종합계획 2016 ~ 2020(The 4th comprehensive plan for national health promotion 2016 ~ 2020)	2016(2016–2020)	All people build and share a healthy world
Republic of Korea	Health plan 2030 for national health promotion (the 5th national health plan (HP2030))	2020(2020–2030)	Increase the healthy life expectancy and improve health equity
Russia	Плaн дeятeльнocти Mиниcтepcтвa здpaвooxpaнeния Poccийcкoй Фeдepaции нa пepиoд c 2016 пo 2021 гoд (Action plan of the ministry of health of the Russian federation for the period from 2016 to 2021)	2016(2016–2021)	The main goal is to ensure access to medical care and improve the effectiveness of medical services, the volume, types and quality of which should correspond to the level of disease and the needs of the population, advanced achievements of medical science
Russia	Russian Federation: Patient-friendly new model for outpatient clinics is the key to the dual-track response in primary health care	2022	The strategic goals of the policy are to increase the country’s population, improve people’s standard of living, increase life expectancy and improve health
Spain	OECD health policy overview-health policy in Spain	2017	
Spain	Public health strategy 2022 ESP 2022 Improving the health and well-being of the population	2022	It has a broad and integrating vision: surveillance, prevention, promotion, health protection, foreign and international health, information systems, research and training in public health, incorporating the gender and equity perspective in all public health actions. In short, it is the tool to contribute to its primary objective, which is to improve the level of health and well-being of our population
Switzerland	Health2030 – the Federal council’s health policy strategy for the period 2020–2030	2020(2020–2030)	People in Switzerland live in an environment that is conducive to health, regardless of their state of health and socio-economic status. They benefit from a modern and high-quality and financially sustainable health system
Switzerland	Endemiestrategie Covid-19 + Strategie zur Verhütung und Bekämpfung von Covid-19 und anderen viralen respiratorischen Krankheiten	2023	The health burden on the population, especially people at particular risk, caused by SARS-CoV-2 and other respiratory viruses is reduced and as a result caused overload of the health system
UK	PHE strategy 2020–25	2019(2020–2025)	Keeping people safe, preventing poor health, narrowing the health gap and supporting a strong economy
UK	NICE strategy 2021 to 2026	2021(2021–2026)	To improve health and wellbeing by putting science and evidence at the heart of health and care decision making
USA	2018–2022 HHS strategic plan	2018(2018–2022)	The mission of the U.S. Department of Health and Human Services (HHS) is to enhance and protect the health and well-being of all Americans by providing for effective health and human services and by fostering sound, sustained advances in the sciences underlying medicine, public health, and social services
USA	HHS strategic plan, FY 2022 – 2026	2021(2022–2026)	The mission of the U.S. Department of Health and Human Services (HHS) is to enhance the health and well-being of Americans, by providing for effective health and human services and by fostering sound, sustained advances in the sciences underlying medicine, public health, and 86 social services
USA	Health people 2020	2010(2010–2020)	A society in which all people live long, healthy lives
USA	Healthy people 2030	2020(2020–2030)	A society in which all people can achieve their full potential for health and well-being across the lifespan

Columns with grey background are NHPSPs after COVID-19

### Themes or priorities

Within the included NHPSPs of selected countries, more than half of 18 pre-COVID-19 NHPSP documents included a theme or priority around providing a package of high-quality integrated and people-centered health services (ensuring all people have access to health services that are coordinated around their needs, respect their preferences, and are safe, effective, timely, affordable, and of acceptable quality, 55.6%) and promoting and protecting the health of communities and public health (focus on prevention and creating safe, resilient, sustainable, and healthy communities, 88.9%). Post-COVID-19, some new themes or priorities besides the above two became mainstream, with more than half of the documents covering theme or priority domains of promoting and protecting the health of communities and public health (focus on prevention, 94.4%), promoting innovation in health research, technologies, and products and improving laboratory capacity (through financial support, infrastructure development and interdisciplinary cooperation, 61.1%) and promoting one health (sustainably balancing and optimizing the health of people, animals and ecosystems, 66.7%). Other themes or priorities were less identified. Eight priority domains had a similar frequency of occurrences, which differed by not more than 10%, in pre- and post-pandemic NHPSPs, namely ensuring financial health protection (ensuring prepayment and pooling of resources for health, rather than relying on people paying for health services out-of-pocket at the time of use), promoting the equity of health (everyone can attain their full potential for health and well-being), promoting and protecting the health of communities and public health (focus on prevention), strengthening health information system and health literacy (strengthening the capacity of health system to collect, manage, understand and utilize health information and data), enhancing the capabilities, education, and training of the health workforce (providing investment in education and training of health workers and strengthening the match between education and employment strategies in relation to health systems and population needs are contributing to continuous shortage), enhancing both local and international collaboration, cross-sector collaboration (two or more local or international organizations working together across sectors-industry, nonprofit, and government-to achieve mutually beneficial outcomes), promoting environmental health (ensuring clean air, stable climate, adequate water, sanitation and hygiene, safe use of chemicals, protection from radiation, healthy and safe workplaces, sound agricultural practices, health-supportive cities and built environments, and a preserved nature) and establishing monitoring, evaluation and revising mechanisms (allowing for the ongoing review, analysis and understanding of the performance of a NHPSP through its life and continuous improvement).

In contrast, six priority domains showed significantly different frequencies in pre- and post-pandemic NHPSPs. Notably, occurrences of themes such as enhancing surveillance and infectious disease control (monitoring, identifying emerging threats, and implementing effective interventions, 22.2% pre-pandemic vs. 50.0% post-pandemic) and building capacity for health emergencies and crises (researching, preventing, and managing epidemic and pandemic-prone diseases, strengthening detection and response systems, 22.2% vs. 44.4%) more than doubled in post-pandemic NHPSPs (see Table 2, Supplementary Tables 1 and 2).

**Table 2:** Frequencies of occurrence of themes or priorities identified in the NHPSPs of selected countries before and after COVID-19

	Theme or priority domain	Ensuring financial health protection (%)	Providing package of high-quality integrated and people-centered health services (%)	Promoting the equity of health (%)	Building capacity to deal with health emergencies and crisis (%)	Enhancing health system governance (%)	Promoting and protecting the health of communities and public health (focus on prevention) (%)	Promoting innovation in health research, technologies and products; Improving laboratory capacity (%)
OECD countries	Occurrence (pre-COVID)	46.7	46.7	46.7	20.0	33.3	86.7	33.3
	Occurrence (post-COVID)	53.3	66.7	53.3	46.7	53.3	93.3	60.0
BRICS countries	Occurrence (pre-COVID)	66.7	100.0	66.7	33.3	33.3	100.0	100.0
	Occurrence (post-COVID)	0.0	100.0	33.3	33.3	33.3	100.0	66.7
All	Occurrence (pre-COVID)	50.0	55.6	50.0	22.2	33.3	88.9	44.4
	Occurrence (post-COVID)	44.4	72.2	50.0	44.4	50.0	94.4	61.1

	Theme or priority domain	Strengthening health information system and health literacy (%)	Enhancing the capabilities, education, and training of the health workforce (%)	Enhancing both local and international collaboration, cross-sector collaboration (%)	Enhancing surveillance and control of infectious diseases (%)	Promoting environmental health (%)	Establishing monitoring, evaluation and revising mechanisms (%)	One Health (%)
OECD countries	Occurrence (pre-COVID)	26.7	40.0	26.7	26.7	33.3	20.0	46.7
	Occurrence (post-COVID)	46.7	53.3	40.0	46.7	46.7	20.0	73.3
BRICS countries	Occurrence (pre-COVID)	66.7	66.7	33.3	0.0	33.3	33.3	66.7
	Occurrence (post-COVID)	0.0	0.0	0.0	66.7	0.0	0.0	33.3
All	Occurrence (pre-COVID)	33.3	44.4	27.8	22.2	33.3	22.2	50.0
	Occurrence (post-COVID)	38.9	44.4	33.3	50.0	38.9	16.7	66.7

Columns with grey background are NHPSPs after COVID-19

### The weighting of themes or priorities

Although eight domains had similar frequencies of occurrence before and after COVID-19 across all NHPSPs, pre-and-post-pandemic NHPSPs emphasized them differently. The most commonly addressed theme or priority domain across all NHPSPs was promoting and protecting the health of communities and public health (focus on prevention), with 26 documents identifying this domain as a primary theme. Compared to the pre-pandemic NHPSPs, two-thirds of post-pandemic NHPSPs of OECD countries, namely ten documents (Ireland, Australia, France, Korea, Czech, Luxembourg, USA, Spain, New Zealand, and the UK.), put increased emphasis on the eight domains except for the domain of establishing monitoring, evaluation and revising mechanisms, among which the domain increased the most was promoting One Health (see Table 3). However, none of the post-pandemic NHPSPs of BRICS countries showed this pattern (see Table 4).

**Table 3:**
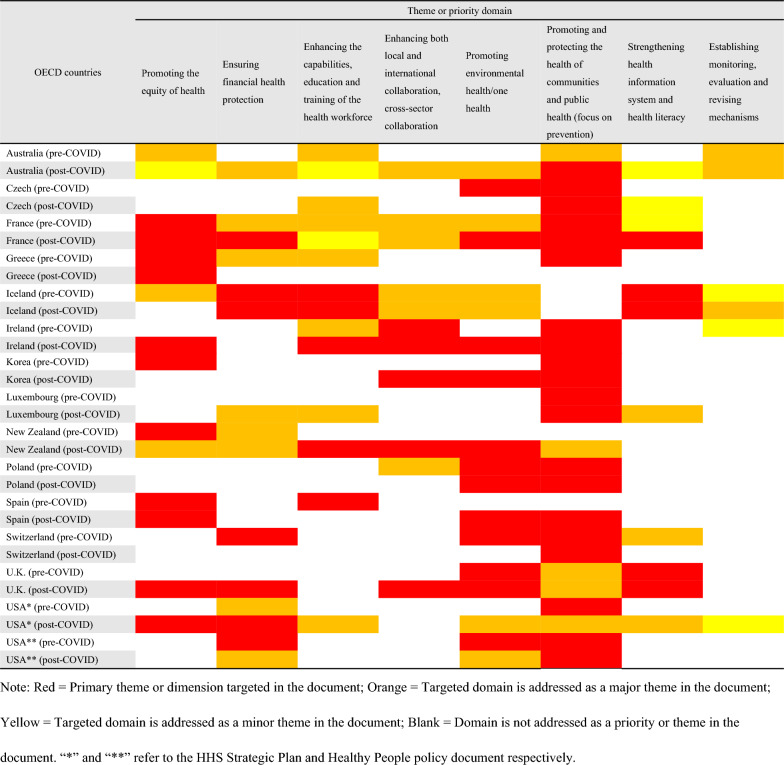
The weighting of themes or priorities that had the same occurrence in NHPSPs before and after COVID-19 in OECD countries

**Table 4:**
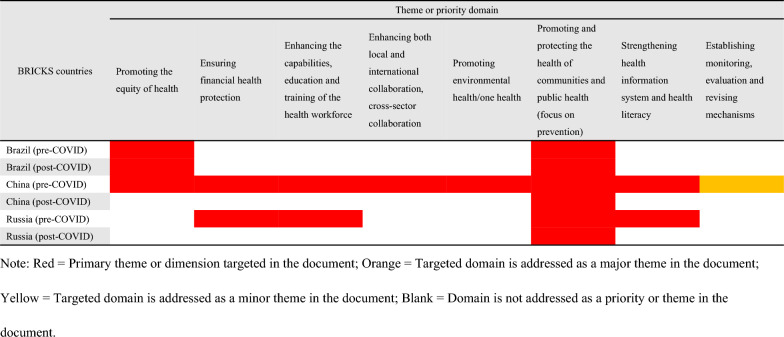
The weighting of themes or priorities that had the same occurrence in NHPSPs before and after COVID-19 in BRICKS countries

### Increased themes or priorities

The contents of theme or priority domains that showed significantly increased occurrence frequency after COVID-19 of post-pandemic NHPSPs were analyzed, and 13 common focuses of sub-themes were identified, including promoting lifelong prevention, improving primary health care, ensuring health services for key groups, developing early warning surveillance systems, developing national health emergency preparedness and response plan, reinforcing leadership and management, ensuring clarity around roles and responsibilities, promoting sectoral coordination, developing and promoting health research and technologies, supporting researchers, enhancing surveillance and control of infectious diseases, improving vaccination and strengthening notification of infectious diseases. More than half of the post-pandemic NHPSPs of OECD countries focused on the domains of improving primary health care (86.7%) and developing and promoting health research and technologies (73.3%) (see Table 5). All the post-pandemic NHPSPs of BRICS countries focused on improving primary healthcare. However, none of the documents of the BRICS countries focused on supporting researchers and improving vaccination (see Table 6).

**Table 5:** Common focuses of theme or priority domains, with increased occurrences after COVID-19, of OECD countries’ NHPSPs after COVID-19

Theme or priority domain	Common focus	OECD														
		Australia	Czech	France	Greece	Iceland	Ireland	Korea	Luxembourg	New Zealand	Poland	Spain	Switzerland	U.K	USA*	USA**
Providing package of high-quality integrated and people-centered health services;	Promoting lifelong prevention		√	√					√			√			√	
	Improving primary healthcare		√		√	√	√		√					√	√	√
	Ensuring health services for key groups					√			√			√				√
Building capacity to deal with health emergencies and crisis	Developing early warning surveillance systems	√					√					√	√			
	Developing national health emergency preparedness and response plan				√		√					√			√	
Enhancing health system governance	Reinforcing leadership and management	√					√			√					√	
	Ensuring clarity around roles and responsibilities			√	√	√	√									
	Promoting sectoral coordination	√			√	√	√									
Promoting innovation in health research, technologies and products; Improving laboratory capacity	Developing and Promoting health research and technologies	√	√	√		√	√		√	√				√	√	
	Supporting researchers	√	√	√			√		√	√					√	
Enhancing surveillance and control of infectious diseases	Enhancing surveillance and control of infectious diseases						√				√	√	√		√	√
	Improving vaccination						√					√				√
	Strengthening notification of infectious diseases						√				√	√	√		√	

“*” and “**” refer to the HHS Strategic Plan and Healthy People policy document respectively

**Table 6:** Common focuses of theme or priority domains, with increased occurrences after COVID-19, of BRICS countries’ NHPSPs after COVID-19

Theme or priority domain	Common focus	Brazil	China	Russia
Providing package of high-quality integrated and people-centered health services;	Promoting lifelong prevention		√	√
	Improving primary healthcare	√	√	√
	Ensuring health services for key groups		√	
Building capacity to deal with health emergencies and crisis	Developing early warning surveillance systems		√	
	Developing national health emergency preparedness and response plan		√	
Enhancing health system governance	Reinforcing leadership and management		√	
	Ensuring clarity around roles and responsibilities		√	
	Promoting sectoral coordination		√	
Promoting innovation in health research, technologies and products; Improving laboratory capacity	Developing and Promoting health research and technologies	√	√	
	Supporting researchers			
Enhancing surveillance and control of infectious diseases	Enhancing surveillance and control of infectious diseases		√	√
	Improving vaccination			
	Strengthening notification of infectious diseases		√	

### The comparison of OECD and BRICS countries

14 out of 38 OECD countries and 3 out of 5 BRICS countries introduced or revised new NHPSP after COVID-19 in OECD countries. More than half of OECD countries identified the domain of promoting and protecting the health of communities and public health as a priority in both pre-and post-pandemic NHPSPs (86.7% and 93.3%). Besides the above domain, more domains identified as priorities by more than half of BRICS countries included providing a package of high-quality integrated and people-centered health services (100%) and promoting innovation in health research, technologies, and products, and improving laboratory capacity (100% and 66.7%) (see Table 2). Compared with pre-pandemic NHPSPs, post-pandemic NHPSPs of OECD countries showed increased occurrences of building capacity to deal with health emergencies and crises (20.0% versus 46.7%) and promoting innovation in health research, technologies and products and improving laboratory capacity (33.3% versus 60.0%) and put increased emphasis on promoting One Health (see Tables 2,  3). In contrast, an increased occurrence of enhancing surveillance and control of infectious diseases (0% versus 66.7%) was only observed in BRICS countries (see Tables 2, Table 4). In addition, the proportion of countries that focused on the domains of supporting researchers in OECD countries far exceeds that in BRICS countries (46.7% versus 0) (see Table 5). On the contrary, BRICS countries are more likely than OECD countries to focus on promoting lifelong prevention (66.7% versus 33.3%) and improving primary health care (100% versus 53.3%) (see Table 6).

### Policy background and Implementation mechanism

**Fig. 2:**
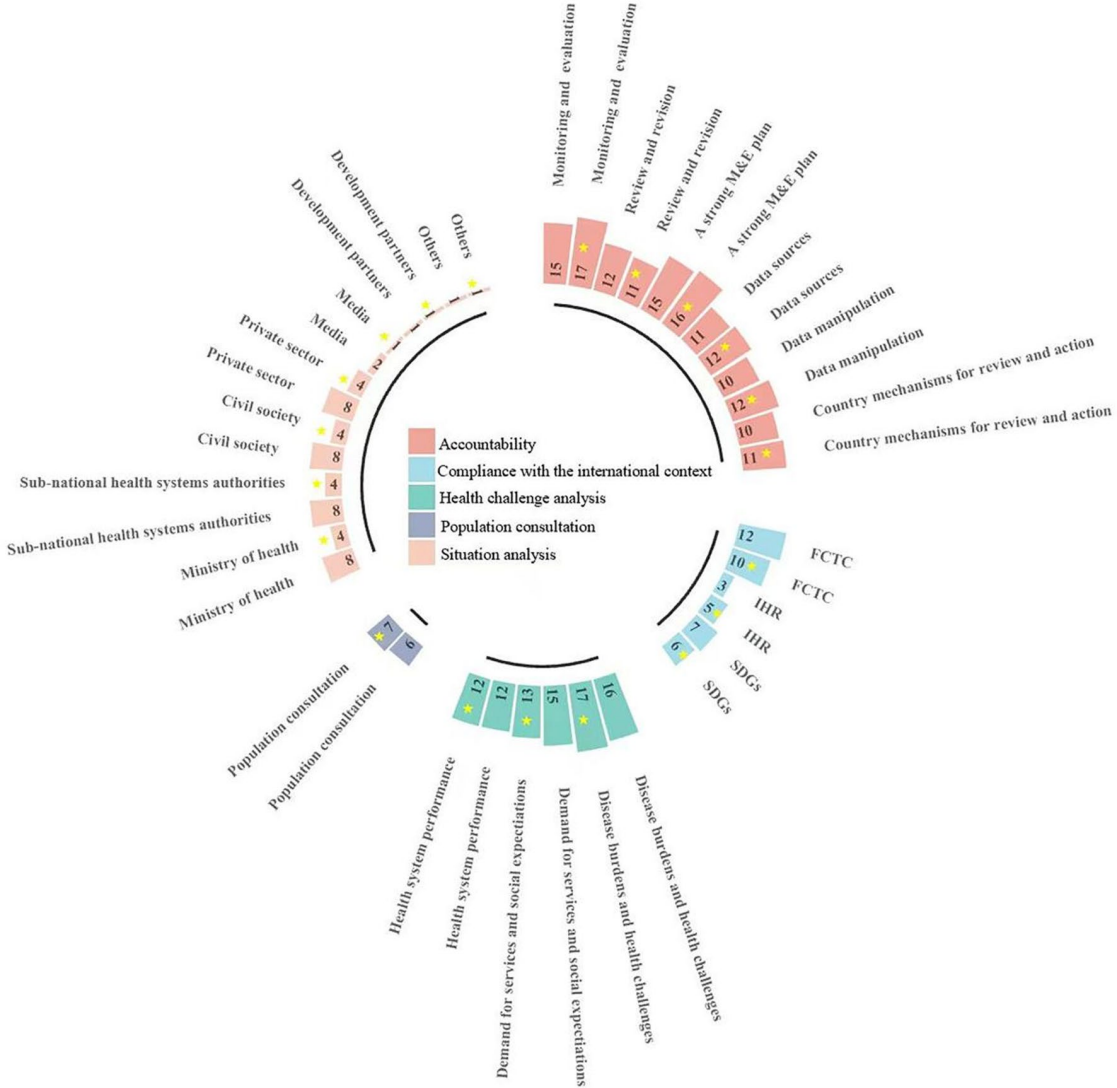
Background and implementation mechanism of NHPSPs of selected countries before and after COVID-19. *Note*: FCTC: Framework Convention on Tobacco Control; IHR: International Health Regulations (2005); SDGs: Sustainable Development Goals.* Note*: Five domains presented were identified according to the Strategizing national health in the twenty-first century: a handbook made by WHO. Four domains, including health challenge analysis, compliance with international context, population consultation, and situation analysis, referred to the policy background. The other domain accountability referred to the implementation mechanism. Blocks with a yellow star refer to post-pandemic documents

Five domains were compared between pre-and-post-pandemic NHPSPs to highlight the variations in background factors (including health challenge analysis, compliance with international context, population consultation, and situation analysis) and implementation mechanism (specifically accountability) of NHPSPs of selected countries (see Fig. 2). Furthermore, selected countries in our analysis seemed to show slightly increasing attention to implementation mechanisms, including monitoring and evaluation of NHPSPs, capabilities of data manipulation, and data sources. Besides these areas, researchers did not find many significant frequency changes in other domains due to COVID-19.

## Discussion

### Main findings

The analysis systematically examines the contents of NHPSPs of OECD and BRICS countries and the structural changes demarcated by the COVID-19 pandemic. It was found that a majority of countries demonstrated common priorities, such as improving health service quality, focusing on prevention, and enhancing collaboration both locally and internationally. Most post-pandemic NHPSPs became more comprehensive, presenting newly established or increased emphasis on many domains. For instance, the frequency of themes related to infectious disease surveillance and control, as well as resilience to health crises, more than doubled. Notably, OECD countries tended to make broader strategic changes after COVID-19, while BRICS countries only showed increased emphasis on surveillance and control of infectious diseases and resilience to health crises. Regarding the background of the NHPSP documents, there was not much variation except for increasing compliance with the International Health Regulations (2005). Our findings fill the gap in identifying and understanding strategic response to the worldwide health crisis—COVID-19 and highlight the importance of building a robust and agile health system to prepare for future health challenges.

### Infection control and resilience to crises

Practical action plans for infection prevention and control (IPC) and health system resilience towards emerging health threats are crucial in the post-pandemic era. WHO’s recommendations on IPC call for Member States strengthen IPC measures, which contributes to reducing enormous avoidable deaths, reducing health costs, providing safer healthcare, and achieving robust health systems [22–24]. However, our study suggests that only about half of the countries specifically nominated prevention and control of infectious diseases and building capacity to deal with health emergencies and crises as health system priorities following COVID-19. The proportion indicates an increasing but still insufficient emphasis on establishing a more resilient and responsive health system.

The implementation of recommendations for optimal practice often encounters significant barriers. A comparative health policy analysis in Europe demonstrated many countries might fail to follow the established optimal practice owing to barriers such as limited political will, government effectiveness, economic constraints and cultural differences [25]. This is in accordance with WHO's first-ever global report on IPC that stated that despite the encouraging progress in IPC globally, only four out of 106 assessed countries (3.8%) had all minimum requirements for IPC in place at the national level during 2021–2022 [26].

Overall, the included NHPSPs mainly listed the following objectives under this theme: strengthening surveillance and early detection of infectious diseases, strengthening preparedness and response to health emergencies, enhancing information systems and the communication and accuracy of epidemiology data, and ensuring close coordination between all sectors as well as the community. Accordingly, the specific action plans outlined to achieve these aims focused on system strengthening and service delivery, such as improving laboratory capacity of pathogen testing, enhancing the reporting system of registered infections, case management and antimicrobial resistance, and popularizing vaccination. These derived NHPSPs also align with WHO-recommended interventions for infection prevention and control [26]. The consistency between our analysis and WHO initiatives underscores the critical importance of investing in and implementing measures for emergency prevention and management, emphasizing the essential role of proactive health system strengthening.

### One health

The WHO has defined one health as "an integrated, unifying approach that aims to sustainably balance and optimize the health of people, animals, and ecosystems" [27]. The pandemic is a one health issue that highlights both the interdependence of human health, animal health, and environmental health and the need for an interdisciplinary vision to produce fundamental and comprehensive scientific and epidemiological knowledge [28]. Our study suggests that a majority of OECD countries but none of the BRICS countries put increased emphasis on one health after the COVID-19 outbreak. The finding indicates an increasing awareness of the critical importance of one health in OECD countries but was relatively inadequate in BRICS countries, which could also be attributed to limited sample size and requires further exploration.

One Health supports a comprehensive approach to disease control, addressing the full spectrum from prevention and detection to preparedness, response, and management. It contributes to global health security by fostering cross-sector and interdisciplinary collaboration, essential for managing complex zoonoses, such as the highly pathogenic avian influenza (HPAI) subtype H5N1 in 2004 and the recent COVID-19 pandemic, which posed significant global health threats [27–30]. Our study highlights the importance of promoting a vision of one health and formulating a corresponding action plan.

### The NHPSP priorities comparison of OECD and BRICS countries

Promoting and protecting public health is crucial for countries to ensure that the populations are healthy and achieve positive states of well-being [26]. Our study suggests that NHPSPs of both OECD and BRICS countries treated public health promotion and protection as a central theme in both the pre-and-post-pandemic eras, which aligns with the significance of the theme. While the BRICS countries, as a collective, demonstrate a greater focus on health service provision and health technology innovation compared to OECD nations [31], it is crucial to recognize the economic disparities within the bloc. Brazil and South Africa, for instance, experience more pronounced resource constraints, whereas China and Russia, with their relatively advanced economies, possess a greater capacity for investment in health technology. This diversity suggests that not all BRICS countries can be uniformly classified as developing nations. Nonetheless, they share common health challenges, such as limited health expenditures, substantial disease burdens, and inequitable access to services. The specific nature and extent of these challenges are shaped by each country's unique economic and historical context [32–34]. Compared to OECD countries, which represent advanced economies, BRICS countries are emerging economies with higher growth rates, although they currently lag in technological development and economic strength [35, 36]. Given these dynamics, health policies in both OECD and BRICS countries may evolve in distinctive ways in the future, meriting ongoing analysis.

### NHPSP development after COVID-19

NHPSPs provide a framework for addressing a wide range of health issues and are essential in protecting and promoting population health. The development of NHPSPs is shaped by each country's political, historical, and socio-economic conditions, making it a complex and dynamic process [37]. Different countries need to develop different NHPSPs according to their contexts. For instance, least-developed and fragile countries with weak health systems and limited fiscal space focus on strengthening foundational health systems, whereas countries with mature health systems may prioritize transformational changes [19].

Our study suggests that only 14 out of 38 OECD countries and 3 out of 5 BRICS countries developed NHPSPs after COVID-19. Countries around the world have taken measures to combat the COVID-19 pandemic. Although countries globally have taken measures to combat the pandemic, most have yet to update or develop NHPSPs specifically for the post-pandemic era. This may reflect the limited time since the COVID-19 outbreak, with many countries likely in the deliberation and revision phase, indicating a time lag in policy updates rather than policy inertia. The impact of COVID-19 extends beyond that of a typical pandemic; it presents a critical opportunity to reframe health systems to better withstand future crises with increased resilience and robustness. In this context, developing new NHPSPs is essential. Future NHPSPs could consider enhancing surveillance and reporting systems, improving cross-sectoral and international cooperation, encouraging innovation, building a compatible health workforce, and improving emergency preparedness [38, 39].

### Strengths, limitations, and future directions

To our best knowledge, our analysis is the first study that investigates the NHPSP response to COVID-19 globally. Besides, the strength of our study lies in the application of a standardized content analysis approach to revise the selected NHPSP documents, with human bias kept to a minimum. These methods can act as a prospective framework for further analysis of health policy across a larger sample or extend to other specific focuses. Several limitations also exist in our study. Since many included documents were written in non-English languages, there is a potential risk of missing or misinterpreting some information due to the limitations of the translation tools. Furthermore, the sample size of the included NHPSPs was modest since most countries have not published new NHPSPs after the COVID-19 outbreak. The possibility cannot be excluded that some NHPSPs were missed despite an extensive search strategy that used multiple sources. Health action plans or frameworks targeting specific domains were excluded in order to ensure standardization in document selection, which did preclude the inclusion of potentially relevant NHPSPs, especially for OECD and BRICS countries excluded in this study. Also, the disproportionate number of BRICS countries may appear to influence the overall findings. Though they do share common features such as large populations and rapidly developing health systems, this focus may limit the generalizability of the findings to other developing countries with different economic and health profiles. Therefore, future analysis can be conducted with a broader scope so as to better reveal the global policy-changing trend after the COVID-19 pandemic. Third, although the study developed clear criteria for differentiating document types based on the primary purpose and content structure, and implemented an iterative review process with team consensus to help resolve discrepancies, several challenges due to varying formats, diverse terminology and content overlaps might have introduced potential subjective biases. Future research could explore developing and validating standardized frameworks for categorizing policy documents and cross-country terminology mapping across different countries and contexts to facilitate more accurate interpretation and classification of NHPSPs. Additionally, researchers could further identify and analyze specific political, economic, and socio-cultural barriers that hinder the adoption and effective implementation of the established optimal practice and draw lessons from successful examples.

## Conclusions

COVID-19 pandemic has exposed the limitations and weaknesses of many countries’ health systems, which are endeavoring to move towards more robust and resilient health systems by optimizing NHPSPs. A comparative content analysis of 36 NHPSPs before and after COVID-19 in 14 OECD countries and 3 BRICS countries was conducted. Our study suggests that about half of countries prioritized infection control and resilience to crises as health system priorities and put increased emphasis on one health and compliance with the International Health Regulations (2005) (IHR) after COVID-19. These findings underscore the necessity of global health system reforms to ensure effective health protection and promotion. Furthermore, our study provides actionable recommendations for other countries in formulating their NHPSPs. Future analyses should be more comprehensive to better capture global policy trends, providing a valuable reference for post-pandemic NHPSP formulation.

## Supplementary Information

Supplementary material 1.

Supplementary material 2.

## Data Availability

All relevant data are reported in the paper.
