# Supplementary material for: Comparative content analysis of national health policies, strategies and plans before and after COVID-19 among OECD and BRICS countries
Source: Glob Health Res Policy. 2025 Feb 21;10:6. doi: 10.1186/s41256-024-00400-y (PMC11843992; doi:10.1186/s41256-024-00400-y)
Supplement: Supplementary file 1 — Supplementary material 1. [file 41256_2024_400_MOESM1_ESM.docx]

Document ID:

Country ID:

Reviewer name and ID:

Data Extraction Template for NHPSP

(National health policies, strategies or plans)

Section 1: Document characteristics

- 1. Is the document a primary document or secondary document?

• Primary documents are full or stand-alone national or jurisdictional policy or strategy documents. Primary documents may be brief, but should be interpretable as a stand-alone document.

- Secondary documents accompany primary documents (e.g. info-graphics, summary pages, excerpts from primary documents) and do not represent the full policy or document.

Primary secondary neither of these (provide explanation below)

| Explanatory notes: |
| --- |

1.2 Does the document (primary or secondary) describe national health policies, strategies or plans?

yes continue to 1.3

no exclude the document and cease review of this document.

1.3 Policy/document title

| Title in original language:  Title translated to English: |
| --- |

1.4 Country of origin

|  |
| --- |

1.5 Language of original publication

|  |
| --- |

1.6 Publication year

|  |
| --- |

1.7 Planning year(Timespan)

|  |
| --- |

1.8 Publisher organization

| Publisher’s name(in original language):  Publisher’s name(translated to English): |
| --- |

Section 2: Data extraction

**2.1 Policy Background**

2.1.1 Is the NHPSP compliant with the international context?(in principle level)

- The Global Framework Convention on Tobacco Control  yes no not sure
- The International Health Regulations（2005） yes no not sure
- The 2030 Sustainable Development Goals  yes no not sure

2.1.2 Does the NHPSP had enough population consultation before it was issued and when it was implemented (gauging the population’s expectations and opinions on health-related matters, policy design and policy implementation)?( It is to capture the population’s demands, opinions and expectations on health-related matters, in order to improve policy responses. The answer is yes as long as there is a corresponding survey, but the degree will be different in different countries)

 yes not mention

| If yes, provide this here: |
| --- |

2.1.3 Does the NHPSP had enough situation analysis of the health and related sectors before it was issued and when it was implemented ?( with all its strengths, weak- nesses opportunities and threats, including their root causes and effects to give a voice and platform to all health sector stakeholders. A situation analysis can be judged as successful if it adequately captures a broad range of the stakeholders’ views and opinions in a balanced way，If mentioned in the acknowledgement, a formal analysis of the organization can be considered)

- Ministry of health  yes no
- Sub-national health systems authorities  yes no
- Civil society (including professional associations and special interest group)  yes no
- Private sector (pharmacy, health technology)  yes no
- Media  yes no
- Development partners  yes no
- Others  yes no

| If yes, provide this here: |
| --- |

2.1.4 Is the NHPSP analyzes the challenges in the of the following aspects?

- Current and projected disease burdens and health challenges  yes no
- Demand for services and social expectations  yes no
- Health system performance and resources, especially the gaps to health needs  yes no

| If yes, provide this here: |
| --- |

**2.2 Comprehensive, balanced and coherent NHPSP**

2.2.1 Does the NHPSP explicitly state purpose, aim or vision?

 yes no

| If yes, provide this here: |
| --- |

2.2.2 Is UHC as an overarching vision of the NHPSP?

- Leaving no one behind;  yes no
- Ensuring financial health protection;  yes no
- Providing package of high-quality integrated and people-centered health services; (focus on the delivery of health services; The connotation of the people-centered integrated health service system is: taking health as the center, taking health needs as the guide, relying on organizational management innovation and operational mechanism transformation, implementing prevention as the focus, providing primary health service as the core.)

 yes no

2.2.3 Does the policy directions move towards to the following themes?

- The level and equity of health  yes no
- Shifting health-care delivery towards integrated people-centered health services(focus on health system level)

 yes no

- Promoting and protecting the health of communities and public health (focus on prevention)  yes no
- Building capacity to deal with crisis and future challenges  yes no

2.2.4 What are the priorities of the NHPSP? (five key criteria for determining the priorities of NHPSP: burden of the health issue, effectiveness of the intervention, cost of the intervention, acceptability of intervention, fairness)(The question refers to specific priority health issues and also includes aspects of the health system.)

| Provide the priorities here: |
| --- |

2.2.5 Does the NHPSP contain the following contents?

- Delivery of comprehensive health services, including personal and non-personal, clinical and non-clinical services  yes no
- Health systems governance  yes no
- Systems for surveillance and forecasting  yes no
- Health research  yes no
- Laboratory capacity  yes no
- Specific intervention plans  yes no
- Health information system  yes no
- Health workforce strategy  yes no
- The resources required: medical products and technologies, and infrastructure  yes no
- Collaboration/coordination with other sectors  yes no
- Sequencing and timing of the activities  yes no
- Attributing general responsibilities(specific description of responsibilities)  yes no
- Surveillance and control of infectious diseases  yes no

2.2.6 Does the NHPSP content mention the following aspects?

- Cost for the NHPSP  yes no
- Budget of the government for the NHPSP  yes no
- The investment strategy and a strategy for mobilizing the required funds  yes no
- Linking interventions (activities and invest­ments) with resource attribution  yes no

| If yes, provide here in detail: |
| --- |

2.2.7 Is the NHPSP coherent with national development plans or other program - specific plans?

- National development plans  yes no not sure
- Program - specific plans (focus on specific health problems)  yes no not sure

| If yes, provide here in detail: |
| --- |

2.2.8 Have the intersectoral mindset been fostered in the NHPSP, not only among the health sectors but also other sectors beyond health?

- Among the health sectors(including public and private sectors)  yes no not sure
- Other sectors beyond health  yes no not sure

| If yes, provide here in detail: |
| --- |

2.3 Implementation mechanism for NHPSP

2.3.1 Leadership and governance arrangements for implementing the strategy?( including overall plan and specific activities)

 yes no not sure

| If yes, provide here in detail: |
| --- |

2.3.2 Strong accountability, does the mechanism of monitoring, evaluation and review of the NHPSP have been formed?

- Regularly monitoring and evaluation of NHPSP  yes no
- Review and revised the NHPSP  yes no
- A strong M&E plan as an integral component of the national health strategy, a comprehensive logical framework that guides selection of indicators and targets, use of international data standards, unified data architecture and innovations;  yes no
- Well-functioning data sources including civil registration and vital statistics (CRVS) systems, population-based surveys, routine facility information systems, facility surveys;

 yes no

- Strong institutional capacity for data col­lection, management, analysis, use and dissemination;

 yes no

- Effective country mechanisms for review and action;(focus on the mechanisms level)

 yes no
