# Supplementary material for: Comparative content analysis of national health policies, strategies and plans before and after COVID-19 among OECD and BRICS countries
Source: Glob Health Res Policy. 2025 Feb 21;10:6. doi: 10.1186/s41256-024-00400-y (PMC11843992; doi:10.1186/s41256-024-00400-y)
Supplement: Supplementary file 2 — Supplementary material 2. [file 41256_2024_400_MOESM2_ESM.docx]

Supplementary Table 1. Occurrence of themes or priorities identified in the national health strategies of selected countries before and after COVID-19 in OECD countries.

|  | Theme or priority domain | Ensuring financial health protection | Providing package of high-quality integrated and people-centered health services; | Promoting the equity of health | Building capacity to deal with health emergencies and crisis | Enhancing health system governance | Promoting and protecting the health of communities and public health (focus on prevention) | Promoting innovation in health research, technologies and products; Improving laboratory capacity |
| --- | --- | --- | --- | --- | --- | --- | --- | --- |
| OECD countries | Australia 1 |  |  | √ |  |  | √ |  |
|  | Australia 2 | √ |  | √ | √ | √ | √ | √ |
|  | Czech 1 |  | √ |  | √ |  | √ |  |
|  | Czech 2 |  | √ |  |  |  | √ | √ |
|  | France 1 | √ | √ | √ | √ |  | √ | √ |
|  | France 2 | √ | √ | √ | √ | √ | √ | √ |
|  | Greece 1 | √ |  | √ |  | √ | √ |  |
|  | Greece 2 |  | √ | √ | √ | √ | √ |  |
|  | Iceland 1 | √ | √ | √ |  | √ |  | √ |
|  | Iceland 2 | √ | √ |  |  | √ |  | √ |
|  | Ireland 1 |  |  |  |  | √ | √ | √ |
|  | Ireland 2 |  | √ | √ | √ | √ | √ | √ |
|  | Korea 1 |  | √ | √ |  |  | √ |  |
|  | Korea 2 |  |  |  |  |  | √ |  |
|  | Luxembourg 1 |  | √ |  |  |  | √ |  |
|  | Luxembourg 2 | √ | √ |  |  | √ | √ | √ |
|  | New Zealand 1 | √ |  | √ |  |  | √ |  |
|  | New Zealand 2 | √ |  | √ |  | √ | √ | √ |
|  | Poland 1 |  |  |  |  |  | √ | √ |
|  | Poland 2 |  |  |  |  |  | √ |  |
|  | Spain 1 |  |  | √ |  | √ |  |  |
|  | Spain 2 |  | √ | √ | √ |  | √ |  |
|  | Switzerland 1 | √ | √ |  |  |  | √ |  |
|  | Switzerland 2 |  |  |  | √ |  | √ |  |
|  | U.K. 1 |  |  |  | √ |  | √ | √ |
|  | U.K. 2 | √ | √ | √ |  |  | √ | √ |
|  | USA 1 | √ | √ |  |  | √ | √ |  |
|  | USA 2 | √ | √ | √ | √ | √ | √ | √ |
|  | USA (healthy people) 1 | √ |  |  |  |  | √ |  |
|  | USA (healthy people) 2 | √ | √ |  |  |  | √ |  |
|  | Occurrence 1 | 46.7% | 46.7% | 46.7% | 20.0% | 33.3% | 86.7% | 33.3% |
|  | Occurrence 2 | 53.3% | 66.7% | 53.3% | 46.7% | 53.3% | 93.3% | 60.0% |

(Continued)

|  | Theme or priority domain | Strengthening health information system and health literacy | Enhancing the capabilities, education, and training of the health workforce | Enhancing both local and international collaboration, cross-sector collaboration | Enhancing surveillance and control of infectious diseases | Promoting environmental health | Establishing monitoring, evaluation and revising mechanisms | One Health |
| --- | --- | --- | --- | --- | --- | --- | --- | --- |
| OECD | Australia 1 |  | √ |  |  |  | √ |  |
|  | Australia 2 | √ | √ | √ |  |  | √ | √ |
|  | Czech 1 |  |  |  | √ |  |  |  |
|  | Czech 2 | √ | √ |  |  |  |  |  |
|  | France 1 | √ | √ | √ | √ | √ |  | √ |
|  | France 2 | √ | √ | √ |  | √ |  | √ |
|  | Greece 1 |  | √ |  |  |  |  |  |
|  | Greece 2 |  |  |  |  |  |  |  |
|  | Iceland 1 | √ | √ | √ |  |  | √ | √ |
|  | Iceland 2 | √ | √ | √ |  |  | √ | √ |
|  | Ireland 1 |  | √ | √ |  |  | √ | √ |
|  | Ireland 2 |  | √ | √ | √ | √ |  | √ |
|  | Korea 1 |  |  |  |  |  |  |  |
|  | Korea 2 |  |  |  | √ | √ |  | √ |
|  | Luxembourg 1 |  |  |  |  |  |  |  |
|  | Luxembourg 2 | √ | √ |  |  |  |  |  |
|  | New Zealand 1 |  |  |  |  |  |  |  |
|  | New Zealand 2 |  | √ | √ |  |  |  | √ |
|  | Poland 1 |  |  | √ |  | √ |  | √ |
|  | Poland 2 |  |  |  | √ | √ |  | √ |
|  | Spain 1 |  | √ |  |  |  |  |  |
|  | Spain 2 |  |  |  | √ | √ |  | √ |
|  | Switzerland 1 | √ |  |  |  | √ |  | √ |
|  | Switzerland 2 |  |  |  | √ |  |  |  |
|  | U.K. 1 | √ |  |  | √ | √ |  | √ |
|  | U.K. 2 | √ |  | √ |  |  |  | √ |
|  | USA 1 |  |  |  |  |  |  |  |
|  | USA 2 | √ | √ |  | √ | √ | √ | √ |
|  | USA (healthy people) 1 |  |  |  | √ | √ |  | √ |
|  | USA (healthy people) 2 |  |  |  | √ | √ |  | √ |
|  | Occurrence 1 | 26.7% | 40.0% | 26.7% | 26.7% | 33.3% | 20.0% | 46.7% |
|  | Occurrence 2 | 46.7% | 53.3% | 40.0% | 46.7% | 46.7% | 20.0% | 73.3% |

Note: Country names labelled with 1 means the NHPSP of that country before COVID-19, while country name labelled with 2 means post-pandemic documents. Columns with grey background are health NHPSPs after COVID-19.

Supplementary Table 2. Occurrence of themes or priorities identified in the national health NHPSPs of selected countries before and after COVID-19 in BRICS countries.

|  | Theme or priority domain | Ensuring financial health protection | Providing package of high-quality integrated and people-centered health services; | Promoting the equity of health | Building capacity to deal with health emergencies and crisis | Enhancing health system governance | Promoting and protecting the health of communities and public health (focus on prevention) | Promoting innovation in health research, technologies and products; Improving laboratory capacity |
| --- | --- | --- | --- | --- | --- | --- | --- | --- |
| BRICS countries | Brazil 1 |  | √ | √ |  |  | √ | √ |
|  | Brazil 2 |  | √ | √ |  |  | √ | √ |
|  | China 1 | √ | √ | √ | √ | √ | √ | √ |
|  | China 2 |  | √ |  | √ | √ | √ | √ |
|  | Russia 1 | √ | √ |  |  |  | √ | √ |
|  | Russia 2 |  | √ |  |  |  | √ |  |
|  | Occurrence 1 | 66.7% | 100.0% | 66.7% | 33.3% | 33.3% | 100.0% | 100.0% |
|  | Occurrence 2 | 0.0% | 100.0% | 33.3% | 33.3% | 33.3% | 100.0% | 66.7% |

(Continued)

|  | Theme or priority domain | Strengthening health information system and health literacy | Enhancing the capabilities, education, and training of the health workforce | Enhancing both local and international collaboration, cross-sector collaboration | Enhancing surveillance and control of infectious diseases | Promoting environmental health | Establishing monitoring, evaluation and revising mechanisms | One Health |
| --- | --- | --- | --- | --- | --- | --- | --- | --- |
| BRICS | Brazil 1 |  |  |  |  |  |  | √ |
|  | Brazil 2 |  |  |  |  |  |  | √ |
|  | China 1 | √ | √ | √ |  | √ | √ | √ |
|  | China 2 |  |  |  | √ |  |  |  |
|  | Russia 1 | √ | √ |  |  |  |  |  |
|  | Russia 2 |  |  |  | √ |  |  |  |
|  | Occurrence 1 | 66.7% | 66.7% | 33.3% | 0.0% | 33.3% | 33.3% | 66.7% |
|  | Occurrence 2 | 0.0% | 0.0% | 0.0% | 66.7% | 0.0% | 0.0% | 33.3% |

Note: Country names labelled with 1 means the NHPSP of that country before COVID-19, while country name labelled with 2 means post-pandemic documents. Columns with grey background are health NHPSPs after COVID.
